# Refined analytical pipeline for the pharmacodynamic assessment of T-cell responses to vaccine antigens

**DOI:** 10.3389/fimmu.2024.1404121

**Published:** 2024-04-24

**Authors:** Michail Angelos Pavlidis, Nadia Viborg, Mads Lausen, Birgitte Rønø, Daniela Kleine-Kohlbrecher

**Affiliations:** Department of Translational Medicine, Evaxion Biotech, Hørsholm, Denmark

**Keywords:** cell culture, T-cell expansion, immune monitoring, *in vitro* stimulation, antigen specificity, vaccine antigen, pipeline, T-cell response

## Abstract

Pharmacodynamic assessment of T-cell-based cancer immunotherapies often focus on detecting rare circulating T-cell populations. The therapy-induced immune cells in blood-derived clinical samples are often present in very low frequencies and with the currently available T-cell analytical assays, amplification of the cells of interest prior to analysis is often required. Current approaches aiming to enrich antigen-specific T cells from human Peripheral Blood Mononuclear Cells (PBMCs) depend on *in vitro* culturing in presence of their cognate peptides and cytokines. In the present work, we improved a standard, publicly available protocol for T-cell immune analyses based on the *in vitro* expansion of T cells. We used PBMCs from healthy subjects and well-described viral antigens as a model system for optimizing the experimental procedures and conditions. Using the standard protocol, we first demonstrated significant enrichment of antigen-specific T cells, even when their starting frequency *ex vivo* was low. Importantly, this amplification occurred with high specificity, with no or neglectable enrichment of irrelevant T-cell clones being observed in the cultures. Testing of modified culturing timelines suggested that the protocol can be adjusted accordingly to allow for greater cell yield with strong preservation of the functionality of antigen-specific T cells. Overall, our work has led to the refinement of a standard protocol for *in vitro* stimulation of antigen-specific T cells and highlighted its reliability and reproducibility. We envision that the optimized protocol could be applied for longitudinal monitoring of rare blood-circulating T cells in scenarios with limited sample material.

## 1 Introduction

Immune Checkpoint Blockade (ICB) aiming to block inhibitory signals of T-cell activity and invigorate anti-tumor immunity has provided long-term clinical responses in cancer patients. More recently, breakthroughs in computational algorithms have led to an unprecedented improvement of the ability to identify and select neoantigens, cancer-specific antigens arising from non-synonymous genetic alterations. Neoantigen-based personalized cancer vaccines have demonstrated strong and durable clinical responses in cancer patients (1–3). The clinical benefit has been often linked to the frequency and relative potency of neoantigen-specific T-cell responses, which are used as a pharmacodynamic marker (4–15). The advancement of neoantigen-based vaccines has led to the establishment of robust methodologies for monitoring neoantigen-specific T-cell responses in clinical samples. However, the detection and subsequent characterization of rare T-cell subsets is a challenging endeavor that frequently demands specialized techniques to enhance sensitivity. One common approach is the enrichment of antigen-specific T cells. In this study, we validated and optimized an existing protocol for the expansion of antigen-specific T cells using *in vitro* stimulation (IVS) of PBMCs. We used PBMCs from healthy donors with diverse HLA haplotypes and viral epitopes as a model system to evaluate the expansion potential of T cells upon *in vitro* peptide stimulation. Selected viral antigens derived from the CEFT-I pool, which is a collection of defined T-cell epitopes restricted on common HLA class I and II alleles and derive from *Clostridium Tetani*, Epstein-Barr Virus (EBV), Human Cytomegalovirus (HCMV) and Influenza A. Profound validation and qualification steps were undertaken to ensure the sensitivity, specificity, reproducibility, and improved performance of the assays for robust detection of antigen-specific T-cell responses.

Our experiments suggest the potential application of the refined analysis pipeline to longitudinally monitor the functionality of antigen-specific T cells in serially collected blood samples and significantly amplify the numbers of the cells of interest. In the context of human disease, it is a valuable tool for subsequent clinical investigations as it presents a reliable and sensitive method for detecting and characterizing T-cell responses against antigens targeted by personalized neoantigen immunotherapy or for measuring the efficacy of other T-cell-engaging therapies. The rationale behind using the IVS protocol without the inclusion of classical activated T-cell enrichment methodologies following IVS, such as density gradient centrifugation, magnetic bead separation or HLA class I tetramer-based isolation, was to preserve a more flexible and customizable approach to suit the experimental requirements and enhance versatility. Even though these activated T-cell enrichment methods are widely used and validated in immunology research and have been proven effective in enriching activated T-cell populations, they present significant limitations like the high cell input requirement, the need for use of complex instrumentation and, in the case of tetramers, prior need for epitope immunogenicity, often restricted only to HLA class I elements. Generally, both methodologies offer distinct advantages and limitations in detecting rare T-cell population functionalities and should be evaluated and optimized based on the suitability to address the research question. Important aspects here are ensuring consistency across experiments due to variations in stimulation parameters and cell culture conditions.

## 2 Materials and methods

### 2.1 Synthetic peptides

All peptides were synthesized by Biosynth (formerly PepScan, Lelystad, Netherlands) or Genscript (New Jersey, USA) at purity >70%. The lyophilized peptides were dissolved in Dimethyl sulfoxide (DMSO) (Merck, cat. #D8418) to a concentration of 10 mg/ml and used in assays. The sequences of the different peptides used are denoted in **Supplementary Table 1**.

### 2.2 Isolation and cryopreservation of PBMCs

Blood from healthy volunteers was collected by venipuncture in lithium heparin tubes (Greiner Bio-One cat. #455084). Heparinized whole blood was diluted 1:2 in RPMI 1640 (Thermo Fisher Scientific, cat. #11544526) and dispensed on top of Lymphoprep (STEMCELL Technologies, cat. #07851/07861) density medium in a 50 ml LeucoSep tube (Greiner Bio-one, cat. #227290). LeucoSep tubes were centrifuged for 20 min. at 1000 x g. After centrifugation, the plasma layer was carefully removed and the PBMC layer was aspirated and moved to a new centrifuge tube. PBMCs were washed twice in RPMI 1640 with centrifugation for 5 min. at 400 x g between each wash. After the final wash, PBMCs were counted via NucleoCounter (ChemoMetec), centrifuged, and resuspended in freezing medium (FCS + 10% DMSO) and aliquoted into cryovials (Thermo Fisher Scientific, cat. #377267) before transfer to CoolCell boxes (Corning, cat. #479-1848) for controlled freezing in -80°C freezer. Next day, samples were cryopreserved and stored at -150°C until further use. Healthy donors’ PBMCs were HLA genotyped at IMGM Laboratories (Martinsried, Germany).

### 2.3 Melanoma patient PBMCs

Human PBMCs from malignant melanoma patient (case ID #110234) were purchased from BioIVT (West Sussex, United Kingdom). The cells were HLA genotyped at IMGM Laboratories (Martinsried, Germany).

### 2.4 Thawing of cryopreserved PBMCs

Cryopreserved PBMCs were thawed for 10 min. at 37°C. Cells were then transferred to a 15 ml centrifuge tube followed by slow addition of 9 ml pre-warmed X-VIVO 15 medium (Lonza cat. #BE02-053Q) + 5% human serum (HS, Sigma-Aldrich, cat. #H3667-100ML). Cells were centrifuged for 10 min. at 330 x g, resuspended in X-VIVO 15 + 5% HS, counted and cell concentrations were adjusted according to the assay needs.

### 2.5 Enrichment of antigen-specific T cells by *in vitro* stimulation

**Figure 1:**
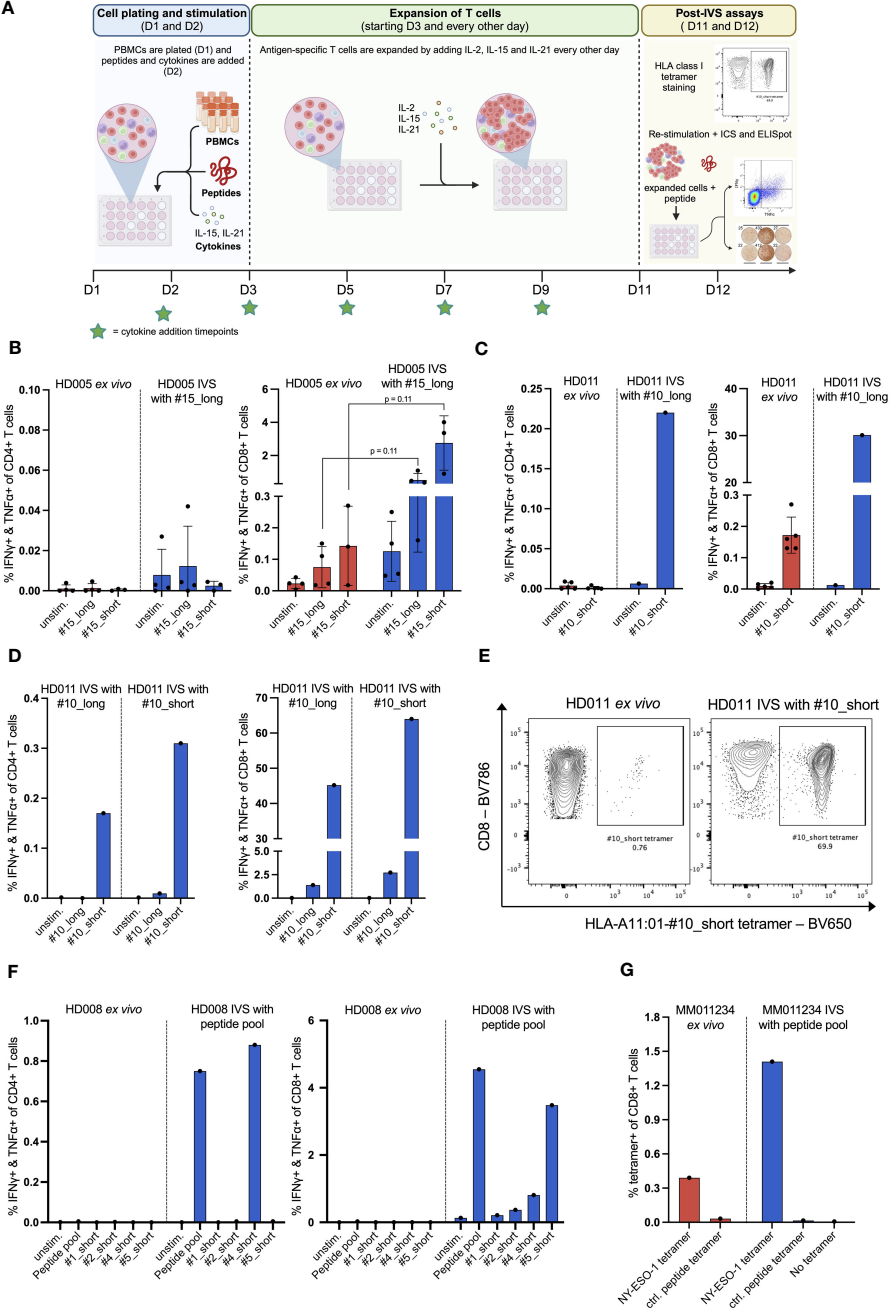
*In vitro* stimulation (IVS) of human PBMCs with peptides following a publicly available protocol leads to strong enrichment of antigen-specific T cells. **(A)** Workflow of the used IVS protocol. Figure created with BioRender. **(B)** % IFNγ+ & TNFα+ within CD4+ or CD8+ T cells upon stimulation of PBMCs from HD005 with peptides #15_long or #15_short either directly *ex vivo* or after IVS with peptide #15_long (n = 4 IVS cultures). *Ex vivo* data come from independent experiments. Each culture was tested in the indicated conditions in a single measurement. One culture was not analyzed for recognition of peptide #15_short. Mean ± SD. A multiple t-test approach was used to test for statistical significance (outlined in “Materials and Methods”) **(C)** % IFNγ+ & TNFα+ within CD4+ or CD8+ T cells upon stimulation of PBMCs from HD011 with peptide #10_short either directly *ex vivo* or after IVS with peptide #10_long (n = 1 IVS culture). *Ex vivo* data come from independent experiments. Mean ± SD. **(D)** % IFNγ+ & TNFα+ within CD4+ or CD8+ T cells upon stimulation of PBMCs from HD011 with peptides #10_long and #10_short after IVS with either of peptides #10_long and #10_short (n = 1 IVS culture per peptide). **(E)** BV650 fluorophore-labelled tetramers consisting of HLA-A11:01 alleles loaded with peptide #10_short were used to stain HD011 PBMCs either directly *ex vivo* or after IVS with peptide #10_short. **(F)** % IFNγ+ & TNFα+ within CD4+ or CD8+ T cells upon stimulation of PBMCs from HD008 with four viral peptides (single and pooled) either directly *ex vivo* or after IVS with the pool of the four viral peptides. **(G)** Fluorophore-labelled tetramers consisting of HLA-A2:01 alleles loaded with minimal epitopes deriving from the melanoma antigens MART-1, NY-ESO-1, MAGE-A3 and gp100 were used to stain PBMCs from MM011234 melanoma patient either directly *ex vivo* or after IVS with the peptide pool (n = 1 IVS culture). As negative control, a non-immunogenic HLA-A2:01 ligand was used.

Antigen-specific T cells were enriched via culturing of PBMC samples with peptides and T-cell cytokines for 10 days (**Figure 1A**). On day 1, PBMCs were thawed, washed, and counted as described above. Then cells were resuspended in X-VIVO 15 + 5% HS to a concentration of 8-12 x 10^6^ cells/ml before distribution of 0.5 ml of cell suspension into wells of a 24-well plate (Corning, cat. #142485), in which cells were rested overnight in an incubator (37°C, 5% CO_2_). On day 2, peptide(s) of choice were diluted in culture medium, consisting of X-VIVO 15 + 5% HS supplemented with the cytokines interleukin (IL)-15 (10 ng/ml, R&D Systems, cat. #247-ILB-005/CF) and IL-21 (50 ng/ml, R&D Systems, cat. #8879-IL-010/CF). Short peptides (8mers or 9mers) were diluted in culture medium to 3.34 μg/ml while long peptides (27mers) were diluted to 10 μg/ml. 0.5 ml of diluted peptides were distributed into culture wells resulting in a new total volume of 1 ml/well, before continued incubation (37°C, 5% CO_2_). On day 3, culture medium was prepared, supplemented with 120 U/ml of IL-2 (R&D Systems, cat. #202-IL-010/CF), before distribution of 0.5 ml medium into each well, resulting in a new total volume of 1.5 ml/well. On day 5, cells were evaluated under the microscope for confluency and emergence of growth centers. Wells with high confluency (90-100% confluency) were split into the appropriate number of daughter wells (1:1.5 split ratio) and X-VIVO 15 + 5% HS was added to reach 1.5 ml/well. Confluency was kept at a minimum of 80% at all times during culture. Culture medium was prepared, supplemented with 160 U/ml of IL-2, before distribution of 0.5 ml medium into each well, resulting in a new total volume of 2 ml/well. On days 7 and 9, cells were evaluated under the microscope for confluency and emergence of growth centers. Wells with high confluency were split into the appropriate number of daughter wells and X-VIVO 15 + 5% HS was added to reach 1 ml/well. Wells that were not split had half of their culture medium removed, thereby reaching 1 ml/well. Culture medium was prepared, supplemented with 80 U/ml of IL-2, before distribution of 1 ml medium into each well, resulting in a new total volume of 2 ml/well. On day 10, cells were collected in centrifuge tubes and washed in 5 ml of X-VIVO 15 + 5% HS medium (without any added cytokines) by centrifugation for 10 min. at 330 x g. Supernatant was poured off and cells were plated back into wells of a 24-well plate after resuspension in 2 ml/well of X-VIVO 15 + 5% HS medium. Followingly, cells were rested by continued incubation (37°C, 5% CO_2_) overnight and analyzed by post-IVS assays the following day.

### 2.6 Peptide re-stimulation and IFNγ enzyme-linked immunosorbent spot (ELISpot)

PVDF membrane plates (Merck Millipore, cat. #MAIP4510) activated with 35% v/v ethanol were coated overnight with 5 μg/ml anti-IFNγ capture antibody (BD, cat. #51-2555KZ). 3-8 x 10^5^ PBMCs were plated per well and stimulated with 5 µg/ml synthetic peptides (diluted in X-VIVO 15 + 5% HS medium) or unstimulated (media only) in a total volume of 200 μl per well. Cells and stimuli were incubated overnight at 37°C and 5% CO_2_ in IVS ELISpot or for 48 hours in *ex vivo* ELISpot, unless otherwise stated. To detect IFNγ secreting cell spots, anti-IFNγ detection antibody (BD, cat. #51-1818KA), streptavidin-HRP enzyme (BD, cat. #557630) and AEC chromogen substrate (BD, cat. #551951) were applied sequentially following the manufacturer’s protocol. ELISpot plates were imaged and IFNγ spots were counted using an ELISpot reader (Cellular Technology, Ltd).

### 2.7 Peptide re-stimulation and intracellular cytokine staining (ICS)

1-2 x 10^6^ PBMCs plated in round bottomed, 96-well culture plates (Corning, cat. #3799) were stimulated with 5 μg/mL synthetic peptides or left unstimulated (media only) in a total volume of 200 μl X-VIVO 15 + 5% HS medium. Protein transport was inhibited by addition of brefeldin A (GolgiPlug, BD #555029) and monensin (GolgiStop, BD cat. #554724) either immediately or two hours after initiation of stimulation with short or long peptides, respectively. Cells were incubated overnight at 37°C and 5% CO_2_. Cells were washed and then surface stained for CD3 (FITC, BD, cat. #561807), CD4 (PE-Cy7, BD, cat. #557852), CD8 (BV786, BD, cat. #563823), and viability dye (GloCell™ Fixable Viability Dye 510, STEMCELL Technologies, cat. #75010) for 30 min. at 4°C. Hereafter cells underwent fixation and permeabilization (eBioscience, cat. #00-8222-49 and #00-8333-56) before being stained intracellularly for IFNγ (PE, BD, cat. #340452) and TNFα (BV650, Biolegend, cat. #502938) for 30 min. at 4°C. Flow cytometry was performed on FACS Celesta (BD) and the frequencies of cytokine-producing CD4+ and CD8+ T cells were determined in FlowJo software (version 10.8.0), see **Supplementary Figure 1** for gating strategy.

### 2.8 HLA class I tetramer staining for detection of antigen-specific CD8+ T cells

Products for generating HLA multimers were purchased from Tetramer Shop (Denmark) or ImmunAware (Denmark) and were treated following manufacturers’ protocols. HLA tetramers from Tetramer Shop consisted of peptide-receptive HLA class I molecules pre-tetramerized via fluorophore-conjugated streptavidin (Tetramer Shop, now part of 10x Genomics, HLA-A02:01 cat. #HA02-070 and HLA-A11:01, cat. #HA11-006) and loaded with peptides at Evaxion Biotech. HLA class I tetramers from ImmunAware were the products of unfolded heavy chain (‘easYmers’, HLA class I types HLA-A02:01 and HLA-A11:01, cat. #1002-01 and # 1018-01, respectively), re-folded with the peptide of interest at Evaxion Biotech, before tetramerization via streptavidin beads conjugated with the flurophores BV605, BV650, BV421, PE and BB515 (BD Bioscience, cat. #563260, #563855, #563259, #554061 and #563259, respectively). Tetramers were tested for proper folding as per manufacturer’s recommendations. Minimal peptides used for HLA tetramers are denoted in **Supplementary Table 1**.

To monitor the presence of antigen-specific CD8+ T cells in PBMCs, the aforementioned HLA class I tetramers were used to stain PBMCs either directly *ex vivo* or following IVS culturing, as described above. 1-2 x 10^6^ PBMCs were plated in round bottomed, 96-well culture plates (Corning, cat. #3799) and incubated with tyrosine kinase inhibitor Dasatinib (STEMCELL Technologies, cat. #73082) for 30 min. at 4°C. Per sample to be stained, 5 μl of each HLA class I tetramer were gathered (i.e. HLA tetramer panel) in an Eppendorf tube and centrifuged at 3300 x g for 5 min. to pellet any protein aggregates. Cells were then incubated with the HLA tetramer panel (5 μl per HLA tetramer) for 15 min. at 37°C. Afterwards, cells were surface stained for CD4 (PE-Cy7, BD, cat. #557852), CD19 (PE-Cy7, BD, cat. #560728), CD14 (PE-Cy7, BD, cat. #562698), CD8 (BV786, BD, cat. #563823) and viability dye (GloCell™ Fixable Viability Dye 510, STEMCELL Technologies, cat. #75010) for 30 min. at 4°C. Finally, cells were acquired on FACS Celesta for determination of HLA tetramer+ CD8+ T cells, see **Supplementary Figure 1** for gating strategy.

### 2.9 Staining of PBMCs for presence of antigen presenting cells

To quantify APCs, PBMCs were surface stained either directly *ex vivo* or after IVS culturing for CD3 (PerCP-Cy5.5, Biolegend, cat. #100328), CD19 (PE-Cy7, BD, cat. #560728), CD14 (BV786, BD, cat. #563698), HLA-DR (BV421, BD, cat. #562804), CD11b (BB515, BD, cat. #564518) and viability dye (GloCell™ Fixable Viability Dye 510, STEMCELL Technologies, cat. #75010) for 30 min. at 4°C. Finally, cells were acquired on FACS Celesta for determination of the different HLA-DR-expressing APC populations, see **Supplementary Figure 2** for gating strategy.

### 2.10 Statistical analyses

GraphPad Prism 9 for Mac OS X was used for graphing, statistical analyses, and tools. Data variation (error bars) represent the standard deviation (SD), unless otherwise stated in the figure legends. A multiple unpaired t-test approach was used to compare means between groups. Welch correction was applied to account for differences in group variances and the Benjamini, Krieger, and Yekutieli method was used to correct for multiple comparisons using a false discovery rate of 1%. Statistical analysis was only performed when data from at least three independent experiments was available.

## 3 Results

### 3.1 *In vitro* stimulation of healthy donor PBMCs leads to strong expansion of antigen-specific T cells

The reliable detection and characterization of rare T-cell populations in human PBMCs frequently requires their enrichment prior to detection. In the present work, we optimized an existing cell culturing protocol for the expansion of antigen-specific T cells based on the *in vitro* stimulation (IVS) of unfractionated human PBMCs with the antigen(s) of interest in the presence of the cytokines IL-2, IL-15, and IL-21 for a period of 10 days (**Figure 1A**) (6). In the first part of the study, we assessed the protocol-specified conditions on their ability to expand antigen-specific T cells. In the next part, we sought to optimize the protocol conditions based on the expansion and yield of antigen-specific T cells. The viral T-cell epitopes used as stimulants were in the format of synthetic peptides and derive from the CEFT-I peptide pool (**Supplementary Table 1**). Viral peptides were selected based on their predicted ability to bind to HLA class I alleles of the donors, whose HLA haplotypes are shown in **Supplementary Table 2**.

We first applied the protocol to evaluate the expansion of antigen-specific T cells by performing IVS of PBMCs from the healthy donor HD005 with peptide #15_long, a 27mer peptide that derives from EBV and bears an HLA class I-restricted 8mer epitope (#15_short) elongated by the naturally occurring amino acids on both ends. We chose to stimulate the cells in IVS with the long peptide as it would presumably allow for expansion of any peptide-reactive CD4+ and CD8+ T cells due to the inclusion of both HLA class I and class II epitopes. *Ex vivo* stimulation of thawed HD005 PBMCs with the long and the short version of peptide #15 in an IFNγ ELISpot assay elicited strong immune responses of similar magnitude (**Supplementary Figure 3A**), while stimulation followed by ICS indicated the induction of polyfunctional CD8+ T-cell responses but no reactive CD4+ T cells (**Figure 1B**). Notably, IVS of PBMCs with peptide #15_long boosted the CD8+ T-cell immunogenicity of both peptides (#15_long: 0,08% vs. 0,52%, p = 0.11; #15_short: 0,14% vs, 2,76%, p = 0.11), with peptide #15_short driving a stronger response than its longer counterpart (**Figure 1B**). Furthermore, IVS-expanded cells yielded low assay background signal and had high viability on the harvest day (mean 96%, range 93,7 – 97%). These data indicate that applying the protocol for *in vitro* stimulation of PBMCs from HD005 led to significant enrichment of CD8+ T cells reactive to the IVS peptide.

To determine if the above-observed T-cell expansion was only specific to HD005 or a general phenomenon, we applied the *in vitro* culturing protocol to stimulate PBMCs from a second donor, HD011, with the viral peptide #10_long that fits the HLA type of this donor. This 27mer peptide also derives from EBV and encompasses an HLA class I-restricted 9mer epitope (#10_short) flanked by the naturally occurring amino acids. *Ex vivo* stimulation of freshly thawed HD011 PBMCs with peptide #10_short followed by ICS elicited a strong response in CD8+ T cells, while no reactive CD4+ T cells were observed (**Figure 1C**). Peptides #10_short and #10_long were shown to be equally immunogenic in an *ex vivo* IFNγ ELISpot assay (**Supplementary Figure 3B**). Impressively, IVS of PBMCs with peptide #10_long followed by re-stimulation with peptide #10_short resulted in a remarkable frequency of polyfunctional CD8+ T cells (**Figure 1C**, right panel), while a polyfunctional CD4+ T-cell response was also elicited (**Figure 1C**, left panel). IVS-expanded cells were highly viable (91,6%). The robust T-cell responses towards peptide #10_short were confirmed through immune analyses of another HD011 culture expanded *in vitro* with peptide #10_long (**Figure 1D**). In line with the above-described data for HD005, when the HD011 PBMCs expanded *in vitro* with peptide #10_long was re-stimulated with the same long peptide, only a feeble CD8+ T-cell response was incited, and no reactive CD4+ T cells were recorded (**Figure 1D**). Overall, these data demonstrate that the protocol-specified culturing conditions allow for strong enrichment of antigen-specific CD4+ and CD8+ T cells from PBMCs of HD011, with their preferential re-activation requiring exposure to the short epitope.

As IVS-expanded PBMCs from HD005 and HD011 were shown to be significantly more responsive to re-stimulation with the short peptide compared to its long counterpart, we turned to investigate whether the length of the stimulating peptide used in IVS can influence the expansion of peptide-specific T cells. To that end, PBMCs from HD011 were stimulated *in vitro* with peptide #10_short, following the above-described IVS protocol. At the end of the culturing period, harvested cells were highly viable (95%), and were then re-stimulated with peptides #10_short and #10_long, followed by ICS. Overall, re-stimulation with either of the two peptides in the post-IVS assay elicited a very similar CD4+ and CD8+ T-cell immunogenicity profile as that recorded in IVS cultures expanded with peptide #10_long (**Figure 1D**), with #10_short being highly reactive in CD4+ and CD8+ T cells, whereas #10_long peptide drove significantly feebler immune responses. The expansion of antigen-specific CD8+ T cells was further confirmed using the HLA class I tetramer staining methodology. Staining of HD011 PBMCs expanded with the peptide #10_short with fluorescently labeled tetramers consisting of tetramerized complexes of HLA-A11:01 molecules loaded with the peptide #10_short (HLA-A11:01-#10_short tetramer) revealed that approximately 70% of CD8+ T cells recognized the short peptide, corresponding to a near 100-fold increase in the *ex vivo* frequency of these cells in freshly thawed PBMCs (**Figure 1E**). Taken together, these data suggest that, at least in the tested donor and assay conditions, the two peptide lengths are interchangeable during IVS, but complete re-activation of the expanded T cells requires direct exposure to the minimal peptide.

To investigate why IVS-expanded cells demonstrated a limited ability to respond to #10_long peptide re-stimulation in the post-IVS assay, HD011 PBMCs stimulated *in vitro* with peptide #10_short were analyzed for the presence of competent Antigen Presenting Cells (APC), which are required for intracellular processing of long antigens prior to surface HLA-presentation. Compared to their *ex vivo* frequency, a big reduction in HLA-DR-expressing CD19+ (B cells) and CD14+ cells (monocytes) was noted after IVS (**Supplementary Figure 4**). These data indicate a significant drop in the frequency of the cellular players mediating peptide processing in the end of the IVS cultures.

In the context of immune monitoring of immunotherapy-treated patients, the scarcity of PBMC samples available as well as the vast number of cancer targets needed to be interrogated for immune recognition often necessitate simultaneous analysis of multiple T-cell specificities. To that end, we sought to investigate whether multiple stimulating peptides can be used simultaneously for IVS. For that, PBMCs from a third donor, HD008, were expanded *in vitro* with a pool of donor-relevant, HLA class I-restricted 9mer peptides (#1, #2, #4 and #5) following the earlier defined IVS protocol. Short peptides were chosen for IVS, as they were shown above to be interchangeable with longer peptides in this step. The Influenza virus (INF)-derived peptides #1_short and #2_short appeared non-immunogenic in *ex vivo* IFNγ ELISpot assay, whereas peptides #4_short and #5_short, originating from EBV and HCMV respectively, induced strong IFNγ responses (**Supplementary Figure 5A**). These peptides were selected to represent a mixture of immunogenic and non-immunogenic targets to simulate a real-life patient analysis setting. Following IVS, cells were harvested, and their viability was assessed to be 97%. Cells were then re-stimulated with the peptide pool as well as with each of the individual peptides, followed by ICS. The data indicated strong CD8+ T-cell reactivity towards the peptide pool, driven predominantly by recognition of the individual peptides #4_short and #5_short and, to a lesser extent, by #2_short, whereas peptide #1_short appeared non-immunogenic (**Figure 1F**, right panel). Interestingly, a robust CD4+ T-cell response against peptide #4_short was also recorded (**Figure 1F**, left panel). *Ex vivo* stimulation of freshly thawed HD008 PBMCs with the peptide pool as well as each peptide individually followed by ICS did not reveal any responses, in contrast to the IFNγ ELISpot data, presumably attributed to the lower sensitivity of the ICS assay.

To further confirm the strong expansion of CD8+ T cells specific to peptide #5_short, IVS-expanded cells were stained with fluorescently labeled tetramers comprising HLA-A02:01 molecules loaded with peptide #5_short (HLA-A02:01-#5_short tetramer). This analysis highlighted the substantial enrichment of CD8+ T cells specific to this peptide following IVS and confirmed the presence of CD8+ T cells specific to this peptide *ex vivo* (**Supplementary Figure 5B**).

As peptide #1_short appeared immunologically inert following IVS of HD008 PBMCs with the viral peptide pool (**Figure 1F**), we turned to investigate whether the observed lack of immunogenicity is attributable to the simultaneous IVS of PBMCs with multiple peptides, which could provoke peptide competition or immunodominance events. To that end, PBMCs from HD008 were subjected to IVS with peptide #1_short alone, followed by interrogation of T-cell reactivity towards peptides #1_short and #1_long. IVS-expanded cells were highly viable (94,9%).The analysis demonstrated similar levels of immune reactivity compared to the setting where peptide #1_short was used for IVS as part of the peptide pool, confirming that the lack of reactivity towards peptide #1_short in the viral pool-expanded cultures is not attributable to the experimental setup but more likely stems from its poor immunogenicity (**Supplementary Figure 5C**).

Finally, we explored whether the IVS protocol can be applied to interrogate PBMCs from a cancer patient for immune recognition of tumor antigens. To that end, commercially purchased PBMCs from an HLA-A02:01+ malignant melanoma patient (MM110234) were stimulated *in vitro* with a pool of four short HLA-02:01-restricted wild-type tumor antigens. These antigens derived from the well-described melanoma antigens MART-1, NY-ESO-1, MAGE-3 and gp100 and have been shown to be widely recognized by T cells, either spontaneously or following immunotherapy (16–19). The sequences of the peptides are given in **Supplementary Table 1**. Upon IVS, expanded cells were highly viable (95,8%). As sample availability was very limited, we assessed CD8+ T-cell recognition of the peptides through direct multiplexed staining of freshly thawed and IVS-expanded PBMCs with fluorophore-labelled HLA class I tetramers comprising HLA-A2:01 molecules loaded with the four minimal peptides and a negative control peptide. Notably, IVS-expanded cells showed enhanced CD8+ T-cell recognition of the NY-ESO-1-derived peptide compared to its *ex vivo* recognition levels (**Figure 1G**), while no signal was detected for the other peptides or the negative peptide before and after IVS.

Taken together, the above-described data demonstrate that the culturing protocol with the specified conditions for IVS of human PBMCs using a single epitope or a pool of multiple epitopes can significantly amplify the numbers of antigen-specific T cells of undetectable or low *ex vivo* frequency and allow for detection of previously undetected T-cell responses, with no apparent competition between the stimulating peptides.

### 3.2 IVS of healthy donor PBMCs expands peptide-reactive T cells specifically

**Figure 2:**
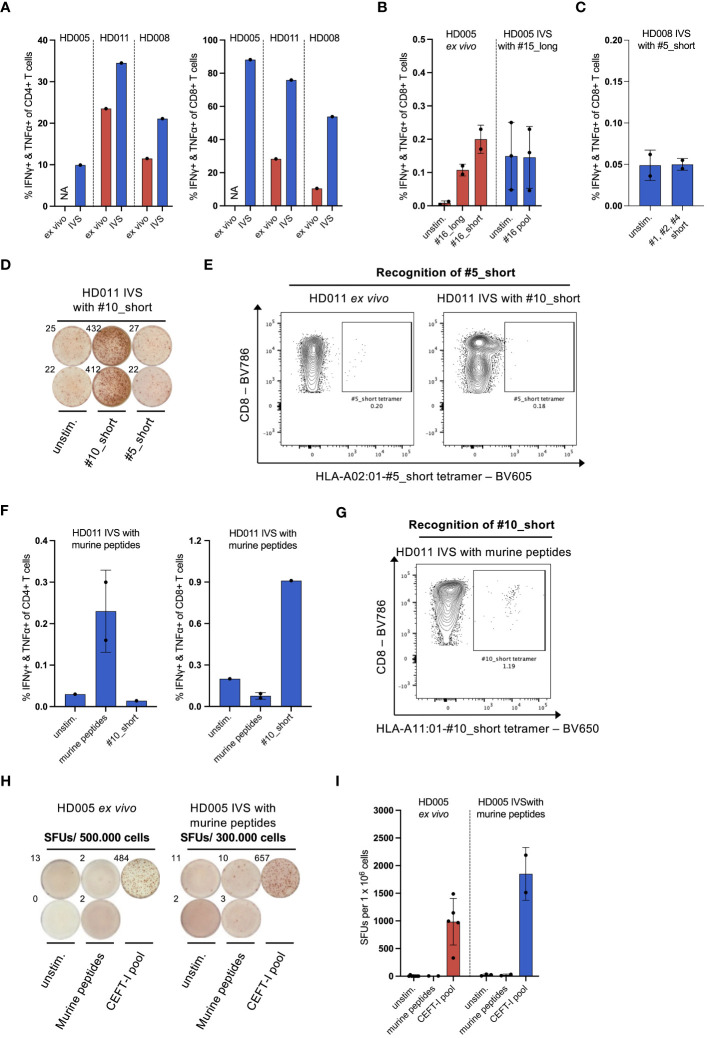
IVS of healthy donor PBMCs specifically enriches T cells reactive to the IVS peptide. **(A)** % IFNγ+ & TNFα+ within CD4+ or CD8+ T cells upon LAC stimulation of PBMCs from the three healthy donors either directly *ex vivo* or after IVS with the viral peptides presented in **Figure 1**. **(B)** % IFNγ+ & TNFα+ within CD8+ T cells upon stimulation of PBMCs from HD005 with peptides #16_long, #16_short or their pool either directly *ex vivo* or after IVS with peptide #15_long (n = 3 IVS cultures). *Ex vivo* data come from one experiment. Each culture was tested in the indicated conditions in a single measurement. Mean ± SD. **(C)** % IFNγ+ & TNFα+ within CD8+ T cells upon stimulation of PBMCs from HD008 with a pool of the short peptides #1, #2 and #4 after IVS with peptide #5_short. (n = 2 IVS cultures). Each culture was tested in the indicated conditions in a single measurement. Mean ± SD. **(D)** Well images from IFNγ ELISpot assay after stimulation of #10_short-expanded HD011 PBMCs with peptides #10_short or #5_short. Top-left numbers indicate the counted Spot Forming Units (SFUs) per 150.000 cells. **(E)** BV605 fluorophore-labelled tetramers consisting of HLA-A02:01 alleles loaded with peptide #5_short were used to stain HD011 PBMCs either directly *ex vivo* or after IVS with peptide #10_short (n = 1 IVS culture). **(F)** % IFNγ+ & TNFα+ within CD4+ or CD8+ T cells upon stimulation of PBMCs from HD011 with a pool of five irrelevant murine peptides or peptide #10_short after IVS with the pool of the murine peptides (n = 1 IVS culture). Murine peptide pool stimulation was tested in technical duplicates. Mean ± SD. **(G)** BV650 fluorophore-labelled tetramers consisting of HLA-A11:01 alleles loaded with peptide #10_short were used to stain HD011 PBMCs after IVS with a pool of five murine peptides (n = 1 IVS culture). **(H)** Well images from IFNγ ELISpot assay and **(I)** extrapolated SFUs per 1 x 10^6^ cells after overnight stimulation of HD005 PBMCs with donor-irrelevant, murine peptides and CEFT-I pool either directly *ex vivo* or after IVS with this pool (n = 1 IVS culture). Top-left numbers in **(H)** indicate the counted SFUs per 500.000 cells (*ex vivo*) or 300.000 cells (IVS). IVS cells was analyzed in two separate experiments. Mean ± SD.

As PBMCs were continuously stimulated with T-cell-promoting cytokines in the course of IVS, we questioned whether T cells contained in the IVS cultures would generally show an increased responsiveness to stimuli. Stimulation of IVS-expanded cells with Leukocyte Activation Cocktail (LAC), an unspecific cell activation mixture, revealed that bulk CD8+ and CD4+ T cells from the above-described IVS cultures were indeed significantly more reactive to general stimulation compared to donor-matched, freshly thawed PBMCs (**Figure 2A**). We thus questioned whether IVS of PBMCs from the three donors could augment the frequency of T-cell populations that recognize antigens that are immunogenic *ex vivo* but not supplemented in the IVS medium. To explore this question, the above-described cultures of HD005 PBMCs stimulated *in vitro* only with peptide #15_long were interrogated for immune recognition of peptides #16_long and #16_short, which derive from EBV and were shown to elicit an *ex vivo* CD8+ T-cell response in ICS of PBMCs from this donor (**Figure 2B**, left panel). Stimulation of peptide #15_long-expanded HD005 PBMCs with a peptide pool comprising peptides #16_short and #16_long did not yield any measurable CD8+ T-cell response (**Figure 2B**, right panel) nor a CD4+ T-cell response (data not shown). Similarly, the above-described cultures of HD008 PBMCs stimulated *in vitro* with peptide #5_short were tested for functional T-cell recognition of a pool comprising the short peptides #1, #2 and #4 shown *ex vivo* to be immunogenic (**Supplementary Figure 5A**). Stimulation with this peptide pool did not yield any CD8+ T-cell responses (**Figure 2C**), indicating the absence of unspecific T-cell expansion. Finally, we expanded this investigation to HD011. To that end, we set up IVS cultures of PBMCs from this donor and stimulated them *in vitro* with peptide #10_short. The strong expansion of T cells specific to peptide #10_short was validated by peptide #10_short re-stimulation in ICS assay and by staining of expanded cells with HLA-A11:01-#10_short tetramer (**Supplementary Figures 6A, B**), highlighting the reproducibility of the assay. We then interrogated the expanded cells for immune recognition of peptide #5_short, which was shown in *ex vivo* IFNγ ELISpot assay to elicit a strong immune response in PBMCs from this donor (**Supplementary Figure 3B**). No immune reactivity against peptide #5_short was recorded in PBMCs expanded *in vitro* with peptide #10_short (**Figure 2D**). To further confirm the absence of immune recognition of peptide #5_short in these IVS cultures, we used HLA-A02:01-#5_short tetramers to measure the CD8+ T-cell recognition of this peptide. In line with the IFNγ ELISpot data, no enhanced recognition of peptide #5_short was detected upon IVS of HD011 PBMCs with peptide #10_short, whereas freshly thawed PBMCs harbored a detectable population of CD8+ T cells specific to #5_short (**Figure 2E**). Taken together, the above-described analyses strongly support the notion that the culturing conditions of the IVS protocol are driving IVS peptide-specific T-cell expansion without leading to measurable increase of bystander T-cell populations present in the starting PBMC material.

As CD8+ T cells reactive to peptide #10_short were heavily expanded in IVS cultures, there is the possibility that any T cells specific to peptide #5_short expanded unspecifically may account for a minute fraction in the IVS culture output, thus rendering their quantification difficult. We thus turned to explore whether expansion of bystander T-cell populations occurs in instances where IVS cultures present more limited CD8+ T-cell enrichment. To that end, we subjected PBMCs to IVS with negative control peptides and interrogated immune reactivity against *ex vivo*-identified immunogenic peptides. We started our analysis with HD011. For that, we performed IVS of HD011 PBMCs with a pool of five donor-irrelevant peptides (predicted *in silico* from the murine CT26 colon carcinoma line) and interrogated the extent that T cells from these IVS cultures recognize the strongly immunogenic peptide #10_short. IVS-expanded cells were highly viable (95,8%). Re-stimulation of the cultures with the pool of the irrelevant peptides only yielded a small CD4+ T-cell response (**Figure 2F**, left panel), signifying that only a minor enrichment of T cells specific to the irrelevant peptides took place. To test T-cell recognition of peptide #10_short, expanded cells were subjected to peptide stimulation followed by ICS as well as direct staining with HLA-A11:01-#10_short tetramers. These two immune analyses revealed a small enrichment of peptide #10_short-specific CD8+ T cells compared to the *ex vivo* levels (**Figure 2F**, right panel and **Figure 2G**).

To explore the phenomenon of bystander T-cell expansion in greater depth, PBMCs from HD005 were stimulated *in vitro* with the pool of five donor-irrelevant murine peptides, as described above, and then tested for functional immune recognition of the CEFT-I pool which encompasses numerous viral targets, including the peptides #15_short and #16_short shown to be highly immunogenic in this donor (**Supplementary Figure 3A** and **Figure 2B**). We chose to use this pool as a means of addressing the recognition of multiple antigen specificities simultaneously. Upon harvesting, the IVS culture showed 92% cell viability. Stimulation of HD005 PBMCs with the irrelevant peptide pool either directly *ex vivo* or after IVS did not yield any responses in IFNγ ELISpot assay, indicating that these peptides are immunologically inert in HD005 (**Figure 2H**). In contrast, IVS of HD005 PBMCs with the irrelevant peptide pool led to an increase of reactivity towards CEFT-I pool compared to *ex vivo* (**Figure 2I**).

Collectively, these data suggest that IVS of human PBMCs strongly favors the enrichment of T cells specific to the IVS peptide, while in cases of feeble or no such enrichment, bystander T cells may undergo an expansion.

### 3.3 IVS workflow can be adjusted to increase the functionality and yield of T cells

**Figure 3:**
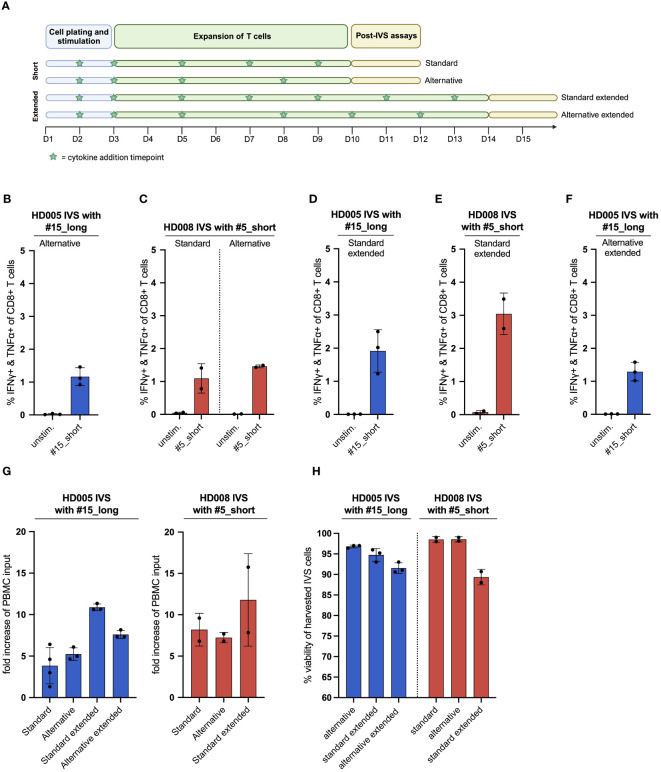
The publicly available IVS protocol can be adjusted to increase the fraction of functional, antigen-specific T cells and the cell yield of the cultures. **(A)** Illustration of the different IVS culturing schemes tested. Figure created with BioRender. **(B)** % IFNγ+ & TNFα+ within CD8+ T cells upon stimulation of HD005 PBMCs with peptide #15_short following IVS with peptide #15_long using the alternative culturing scheme (n = 3 IVS cultures). Each culture was tested in the indicated conditions in a single measurement. Mean ± SD. **(C)** % IFNγ+ & TNFα+ within CD8+ T cells upon stimulation of HD008 PBMCs with peptide #5_short following IVS with peptide #5_short using the standard or the alternative culturing scheme (n = 2 IVS cultures). Each culture was tested in the indicated conditions in a single measurement. Mean ± SD. **(D)** % IFNγ+ & TNFα+ within CD8+ T cells upon stimulation of HD005 PBMCs with peptide #15_short following IVS with peptide #15_long using the extended version of the standard IVS culturing timeline (n = 3 IVS cultures). Mean ± SD. **(E)** % IFNγ+ & TNFα+ within CD8+ T cells upon stimulation of HD008 PBMCs with peptide #5_short following IVS with peptide #5_short using the extended version of the standard IVS culturing timeline (n = 2 IVS cultures). Mean ± SD. **(F)** % IFNγ+ & TNFα+ within CD8+ T cells upon stimulation of HD005 PBMCs with peptide #15_short following IVS with peptide #15_long using the extended version of the alternative IVS culturing timeline (n = 3 IVS cultures). Mean ± SD. **(G)** Fold-increase of the PBMC input numbers after IVS of PBMCs from HD005 and HD008 following the different IVS culturing schemes presented in **Figure 3A**. For the calculation of the fold-increase for standard HD005, the IVS cultures from **Figure 1B** were used. **(H)** % viability of harvested cells in the HD005 IVS cultures (n = 3 IVS cultures per expansion protocol) and HD008 IVS cultures (n = 2 IVS cultures per expansion protocol). Mean ± SD.

Having demonstrated that the IVS protocol-specified culturing conditions drive strong enrichment of antigen-specific T cells with minimal unspecific T-cell expansion, we turned to investigate whether aspects of the protocol could be simplified or adjusted to enhance cell yield. We first assessed whether medium and cytokine replenishment in the IVS expansion phase performed on days 7 and 9 can be alternatively carried out on day 8 (**Figure 3A**). To that end, we performed IVS of freshly thawed PBMCs from HD008 with the response-relevant peptide #5_short following either the protocol-specified timeline with medium and cytokine replenishment on days 7 and 9 (from hereon: standard protocol) or a simpler timeline where the task is performed only on day 8 but added cytokines’ concentration match that of day 7 (from hereon: alternative protocol) (**Figure 3A**). Similarly, we performed IVS of HD005 PBMCs with peptide #15_long following the alternative protocol, as the performance of the standard IVS protocol in HD005 PBMCs expansion with this peptide had already been tested (**Figure 1B**). Upon harvesting, cell viability in the three HD005 “alternative” cultures was above 96%, while the two HD008 “standard” and the two “alternative” cultures had >98% viability (**Figure 3H**). Immune analysis on days 11 and 12 indicated that IVS of HD005 PBMCs with peptide #15_long following the alternative protocol resulted in lower frequency of polyfunctional #15_short-reactive CD8+ T cells, as compared to the standard timeline culture (**Figure 3B**). In contrast, HD008 PBMCs expanded with peptide #5_short following either of the two timelines showed comparable functional CD8+ T-cell recognition of this peptide (**Figure 3C**). Although contrasting data on the influence of the alternative scheme on the attained frequency of peptide-specific CD8+ T cells were obtained in the two healthy donors, both culturing schemes led to noteworthy expansion of peptide-specific T cells compared to their respective *ex vivo* frequency (**Figures 1B, F**).

To test the robustness of the alternative IVS protocol, we gathered longitudinal IFNγ ELISpot data from two different IVS setups performed with PBMCs from HD011 and another donor, HD015, and calculated the inter-assay coefficient of variation (%CV) for each setup. The two assays yielded a similar %CV of around 18% suggesting a high degree of robustness (**Supplementary Figures 7A, B**). Data from five direct *ex vivo* IFNγ ELISpot assays run with HD015 PBMCs stimulated with CEFT-I demonstrate that the ELISpot assay, and not the IVS protocol, has the largest impact on the inter-assay %CV (**Supplementary Figure 7C**).

We then asked whether extending the T-cell culturing phase from 10 to 14 days accompanied by additional cytokine replenishment timepoints on days 11 and 13 could enhance the frequency of functional peptide-specific T cells. To explore this question, freshly thawed PBMCs from the same two healthy donors were subjected to IVS with the same viral peptides described above and underwent medium and cytokine replenishment on days 5, 7 and 9, as described in the standard protocol, and additionally on days 11 and 13 (from here on: standard extended protocol) (**Figure 3A**). On day 14, cells were rested overnight in non-cytokine-containing medium before being analyzed in functional assay. The average cell viability in the three HD005 and the two HD008 IVS cultures expanded using this protocol was 94,7% and 89%, respectively (Figure H). Immunogenicity analyses indicated that, overall, extending the IVS culturing period of HD005 PBMCs did not significantly boost the numbers of functional, peptide-reactive CD8+ T cells compared to shorter IVS culturing (**Figure 3D**). In contrast, extending the IVS of HD008 PBMCs with peptide #5_short led to a notable increase of polyfunctional, peptide-specific CD8+ T cells compared to the 10 day-long culturing (**Figure 3E**). Additionally, culturing the cells for longer time did not increase the assay background levels. Collectively, these data indicate that extending the culturing time of PBMCs preserves or further boosts the frequency of functional peptide-specific CD8+ T cells.

We then turned to investigate whether the extension of the alternative IVS culturing scheme from 10 to 14 days can increase the frequency of functional, antigen-specific T cells. To that end, HD005 PBMCs were stimulated *in vitro* with the response-relevant peptide #15_long following the alternative IVS protocol, with replenishment of the culture medium and the cytokines on day 8 and, additionally, on days 10 and 12 (from here on: alternative extended protocol) (**Figure 3A**). On day 14, cells were rested overnight in non-cytokine-containing medium before being analyzed. The average cell viability in the three HD005 IVS cultures expanded using this protocol was 91,5% (**Figure 3H**). Functionality analysis indicated that culturing the cells for 14 days following the alternative timeline led to comparable CD8+ T-cell responses as the shorter alternative culturing scheme (**Figure 3F**). Taken together, this investigation proposes that extending the alternative culturing timeline may have a minor impact on the frequency of enriched, peptide-specific CD8+ T cells.

Finally, we explored how the different culture schemes tested above influenced the viable cell yield. We found that the standard and alternative IVS culturing schemes perform comparably, leading to a five- to ten-fold increase in the cell output as compared to the assay input PBMC numbers for the two healthy donors, respectively (**Figure 3G**). Notably, extension of the standard and alternative IVS schemes further increased the numbers of harvested cells at the end of the cell culturing.

Overall, the above-presented investigations suggest that elongating the culturing time of *in vitro* stimulated PBMCs significantly increases the viable cell yield and preserves robust functionality of the expanded, peptide-specific CD8+ T cells. Importantly, the IVS assay shows significant reproducibility across different assays runs, an important feature that ensures consistency when analyzing clinical samples.

## 4 Discussion

In the present work, we adapted an existing protocol for IVS of human PBMCs aiming at enriching peptide-specific T cells and optimized technical aspects of it. We first applied this protocol to *in vitro* stimulate PBMCs with short or long versions of peptides harboring characterized HLA class I-restricted epitopes from viruses, accompanied by frequent cytokine replenishment. Through various immune assays, we demonstrated notable amplification of the frequency of cytokine-producing, peptide-specific T cells in three assessed donors following IVS. Across our analyses, IVS cultures were found to have consistently high viability (>90%). These results highlighted that the described methodology for *in vitro* culturing of PBMCs can drive significant enrichment of antigen-specific T cells, even when their starting frequency in PBMCs is below detection limit *ex vivo*.

In these experiments, we first found that the attained frequency of polyfunctional, peptide-specific CD8+ T cells after *in vitro* culturing varied significantly among the analyzed healthy donors. The magnitude of the *ex vivo* immune responses measured in IFNγ ELISpot assay did not completely correlate with the frequency of functional T cells recorded after IVS, suggesting that other factors influence the capacity of T cells to expand *in vitro*. It has been observed that the dynamics of T-cell expansion are vastly influenced by their intrinsic proliferative capacity (20). In this direction, it would be insightful to decipher the phenotype of the viral antigen-specific T cells in the starting PBMC material, as IL-15 used for *in vitro* expansion has been shown to specifically favor memory CD8+ T cells (21).

Next, we observed that re-stimulation of *in vitro* expanded PBMCs with the short peptides induced considerably stronger CD8+ T-cell responses than their long counterparts, even though the latter encompass the minimal epitope and that both peptides were similarly immunogenic *ex vivo*. Additionally, re-stimulation of *in vitro* expanded HD011 PBMCs with the peptide #10_short activated polyfunctional CD4+ T cells, which were however not responsive to stimulation with the long peptide. Interestingly, IFNγ-producing CD4+ T cells targeting 9mer peptides that derive from Wilms Tumor Protein-1 (WT1) and Influenza A have been described before (22, 23). These peptides are believed to bind to HLA class II molecules through peptide anchor motifs present in their sequences. It is well established that peptides from the extracellular space need first to be internalized and processed intracellularly to shorter peptides before the derivative fragments can be loaded on HLA class I molecules and presented on the surface of cells through cross-presentation (24–26). Human PBMCs contain populations capable of presenting antigens, such as monocytes, B-cells and a small number of Dendritic Cells (DCs) (27). Although not scrutinized extensively, we recorded a significant reduction in the numbers of APCs following IVS of HD011 PBMCs. We thus speculate that the scarcity of presentation-competent cells in the IVS cell output underlies the limited responsiveness of expanded cells to long peptides. The presence of these cells in PBMCs is leveraged in other IVS protocols through the provision of agents that promote APC maturation and differentiation to augment the yield of specific T cells, and assay re-stimulation of expanded T cells often requires their co-culture with autologous CD4+ and CD8+ T-cell depleted PBMCs, DCs or B cells loaded with the peptides of interest or transfected with minigenes encoding the relevant antigens (7, 10, 12) (28). In contrast, it is believed that short peptides can circumvent the intracellular antigen processing pathways and bind directly onto surface HLA class I molecules on all nucleated cells, thus allowing for direct activation of CD8+ T cells bearing the cognate T-Cell Receptor (TCR). However, the use of minimal peptides for IVS of PBMCs may limit the ability to study neoepitope-specific CD4+ T-cell responses, which are frequently elicited upon vaccination and hence their detection is of vast importance (4, 7). As the viral peptides used in the presented work were strong CD8+ T-cell inducers, the study of expanded peptide-reactive CD4+ T cells after IVS was limited.

The elicitation of comparable numbers of functional, peptide-specific T cells among HD011 PBMCs cultured in the presence of either peptide #10_short or #10_long suggests that long peptides can be efficiently used in IVS to stimulate CD8+ T-cell responses, likely attributed to the ample presence of presentation-competent cells and the accompanying antigen processing machinery during the culturing phase.

Viral epitopes with defined immunogenicity serve as useful model antigens for thorough protocol validation and optimization; however, immune monitoring of cancer patients often relies on the characterization of responses against weak tumor antigens. In our experiments with the melanoma patient, we chose to stimulate PBMCs *in vitro* with a pool of four tumor antigens representing naturally occurring, non-mutated sequences and are thus more likely to be subject to tolerance. *Ex vivo* HLA class I tetramer staining revealed the existence of a response against NY-ESO-1, which was significantly enriched following IVS. These results showcase the suitability of the IVS protocol for monitoring responses against non-viral but tumor-relevant antigens. In support of this, in-house data have demonstrated the applicability of the IVS protocol for monitoring neoantigen-specific responses, even in undetectable frequency *ex vivo* (data not shown).

Next, we evaluated bystander T-cell expansion in IVS cultures. First, we demonstrated that HD005, HD008 and HD011 PBMCs expanded *in vitro* with immunogenic viral peptides did not respond to re-stimulation with other donor-relevant peptides shown to be immunogenic *ex vivo*. This suggests that the IVS protocol strongly favors the expansion of T cells specific to the immunogenic IVS peptide, thus limiting the unspecific expansion of other T-cell populations present in the cultures. In the next step, we stimulated PBMCs from HD011 and HD005 *in vitro* with a pool of irrelevant donor peptides in order to induce minimal T-cell enrichment and measured the expansion of bystander T cells. In HD011 IVS cultures, we noticed increased CD8+ T-cell recognition of the peptide #10_short compared to *ex vivo*, indicating a small expansion of bystander T cells recognizing this peptide. Interestingly, this irrelevant peptide pool raised a feeble CD4+ T-cell response, presumably attributed to the use of 27mer peptides for *in vitro* stimulation and the presence of immunogenic CD4+ T-cell epitopes. IVS of HD005 PBMCs with the same donor-irrelevant peptides did not expand any cells specific to these peptides but, similarly to HD0011, led to increased recognition of the *ex vivo* immunogenic CEFT-I pool. As this pool represents numerous viral antigen specificities, it would be interesting to assess whether this increase is driven by a dominant T-cell clone (e.g. T cells specific to peptide #15) or multiple clones. As T-cell expansion specific to the irrelevant IVS peptides was small in both cases, the persistence of these bystander T cells may be fueled by high amounts of unconsumed IL-2 that promotes their survival and IL-15, that has been shown to be indispensable for the maintenance of the proliferative capacity of T cells from human peripheral blood (29, 30). Cytokine conditioning of T cells may be underlying the increased responsiveness of IVS-expanded bulk T cells to LAC stimulation known to act through non-specific, TCR-independent pathways. In addition, the used culture medium is supportive of T-cell proliferation (31). The IVS cultures were indeed found consistently to be highly enriched in T cells (99% of live cells were CD3+), even in the instance of HD011 PBMCs expanded with the irrelevant peptide pool. A consequence of this observation is that the significantly higher prevalence of T cells in the IVS cell product compared to *ex vivo* may lead to an overestimation of post-IVS peptide reactivity in the IFNγ ELISpot assay attributable to the plating of a higher number of reactive T cells and not due to true *in vitro* expansion. Consequently, FACS-based assays such as ICS and HLA class I tetramer staining that allow analysis of gated T-cell populations may be better suited to assess the enrichment of antigen-specific T cells. Overall, our data suggest that, when enrichment of T cells specific to the IVS peptide is minimal, bystander T cells may expand. However, the T-cell repertoire of the IVS cell product is likely qualitatively and quantitively different to *ex vivo*, as IL-2-based T-cell expansion methods have been shown to be highly dependent on the *in vitro* growth capacity of the different clones (20). Although the clonality of the IVS-expanded T cells was not extensively studied, our investigations suggest that the IVS peptide provided during cell culturing is largely determining the enrichment of antigen-specific T cells.

In the last part of our analysis, we demonstrated that culturing PBMCs *in vitro* following a modified protocol with fewer cytokine and medium replenishment timepoints preserved strong enrichment of functional, antigen-specific T cells and led to a notable increase in cell yield compared to the input cell numbers. Culture medium and cytokine replenishment every three days or more is common across different IVS protocols (4, 5, 7, 12, 32). Similarly, culturing of PBMCs following an extended version drove notable amplification in the numbers of functional, antigen-specific T cells and further boosted the cell yield compared to shorter culturing schemes. Importantly, these modified versions of the IVS protocol preserved the high cell viability of the IVS cultures. We hypothesize that the increased cell recovery is attributable to the longer time the cells are given to expand. However, it has been reported that the cytokine milieu used for IVS can also greatly influence the IVS cell yield, as a mixture of IL-2, IL-15 and T-cell growth factor (TCGF) was superior to IL-2 alone (32). Together, these optimizations entail greater user flexibility accompanied by reduced intervention in the cultures and, importantly, allow for the application of a greater number of post-IVS investigations, which is pivotal in instances of limited clinical sample availability.

Collectively, the data presented here demonstrate the robustness and specificity of the investigated IVS protocol to expand peptide-specific T cells of low or even undetectable *ex vivo* frequency and suggest ways to maximize the recovery of functional T cells. We envision that the developed pipeline may be reliably applied to measure the functionality of antigen-specific T cells in blood-derived samples longitudinally.

## Data availability statement

The original contributions presented in the study are included in the article/**Supplementary Material**. Further inquiries can be directed to the corresponding author.

## Ethics statement

The studies involving humans were approved by The Regional Ethics Committee in the Capital Region of Denmark (approval number H-23045372). The studies were conducted in accordance with the local legislation and institutional requirements. The participants provided their written informed consent to participate in this study.

## Author contributions

MP: Conceptualization, Data curation, Formal analysis, Investigation, Methodology, Validation, Visualization, Writing – original draft, Writing – review & editing, Funding acquisition. NV: Conceptualization, Data curation, Formal analysis, Investigation, Methodology, Supervision, Validation, Visualization, Writing – original draft, Writing – review & editing. ML: Data curation, Formal analysis, Investigation, Visualization, Writing – original draft, Writing – review & editing, Software. BR: Conceptualization, Funding acquisition, Investigation, Project administration, Resources, Supervision, Writing – original draft, Writing – review & editing, Validation. DK-K: Conceptualization, Funding acquisition, Project administration, Resources, Supervision, Writing – original draft, Writing – review & editing, Validation.

## Supplementary material

Supplementary Table 1 Overview of the used peptides and their features.

Supplementary Table 2 Overview of HLA class I and II alleles of the healthy donors and the melanoma patient.
