# Supplementary material for: Refined analytical pipeline for the pharmacodynamic assessment of T-cell responses to vaccine antigens
Source: Front Immunol. 2024 Apr 24;15:1404121. doi: 10.3389/fimmu.2024.1404121 (PMC11076743; doi:10.3389/fimmu.2024.1404121)
Supplement: Supplementary Table 1 — Overview of the used peptides and their features. [file DataSheet_1.pdf]

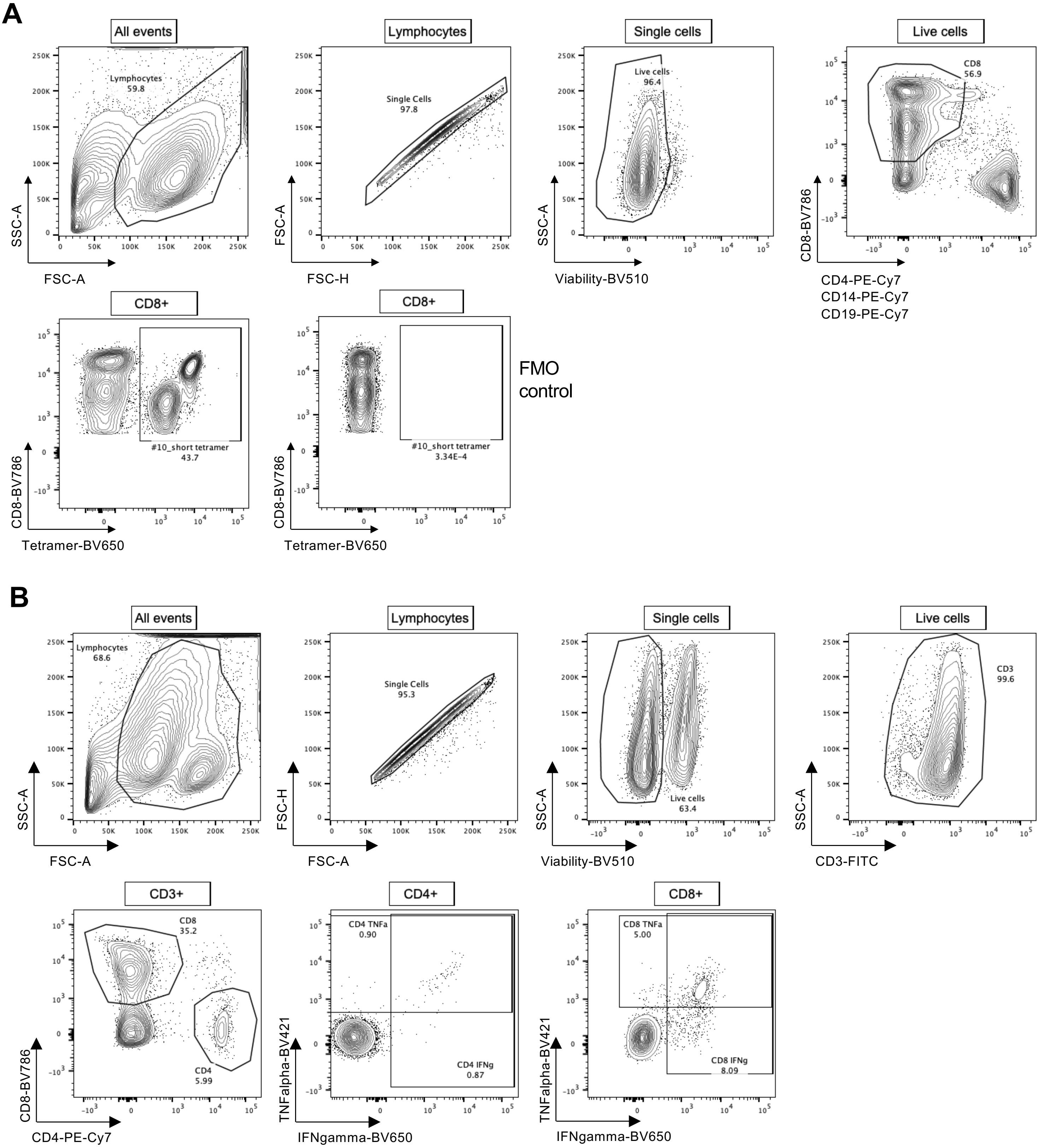

Supplementary Figure 1. FACS gating strategies for HLA class I tetramer staining and peptide re-stimulation followed by ICS. **(A)** To quantify antigen-specific CD8+ T cells, PBMCs were stained either directly *ex vivo* or after IVS with antibodies specific for the markers CD8, CD4/CD14/CD19 (dump), viability dye and fluorescently labelled tetramers comprising the HLA class I alleles HLA-A11:01 or HLA-A02:01 loaded with minimal peptides. Tetramer-positive gate was set based on fluorescence minus one (FMO) control. Example of staining with #10_short tetramer is presented. **(B)** Following stimulation, human PBMCs were stained with viability dye and antibodies specific for CD3, CD4, CD8, IFNγ and TNFα markers. Single cytokine-positive gates were set based on non-stimulated samples.

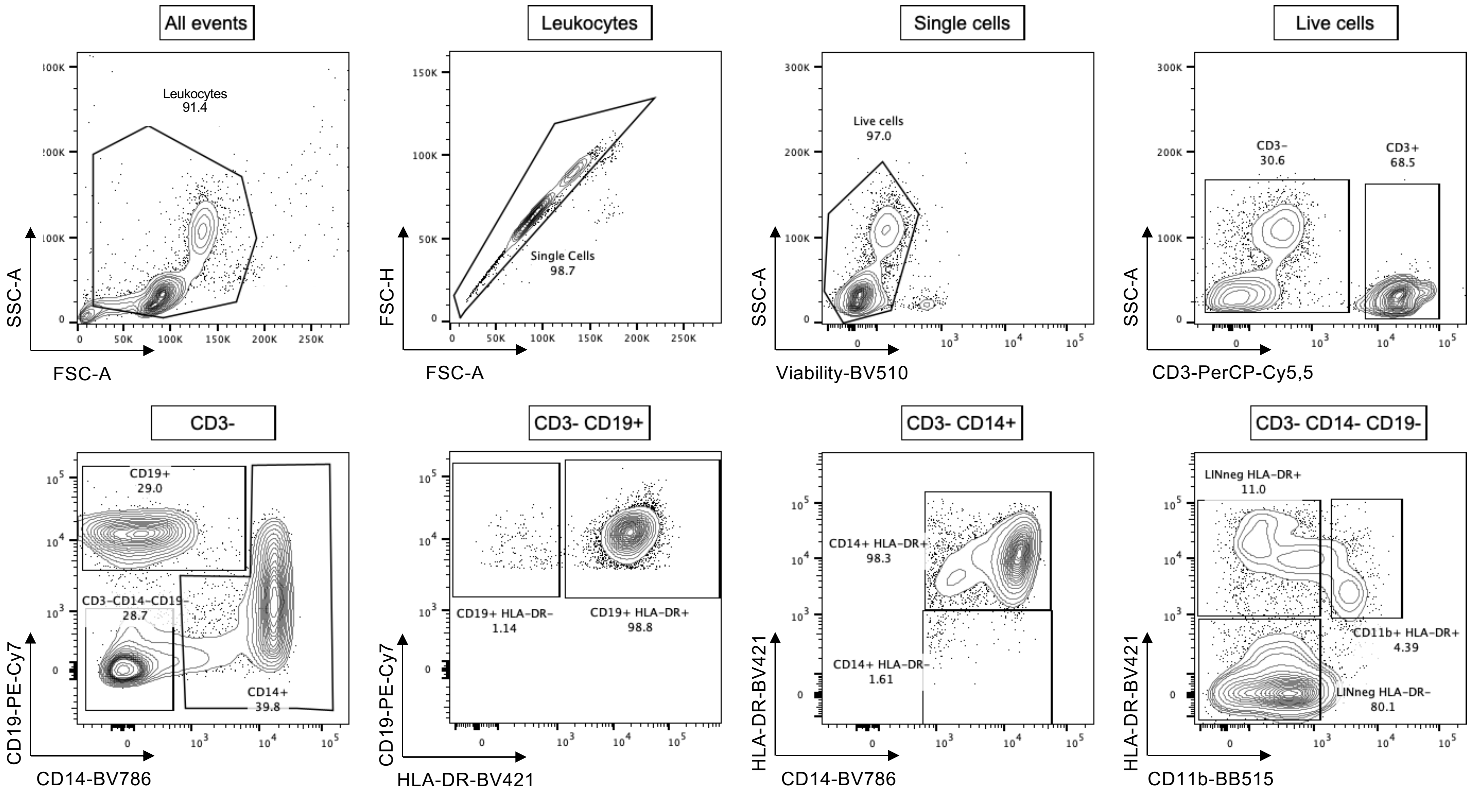

Supplementary Figure 2. FACS gating strategy for APC staining *ex vivo* or after IVS of HD011 PBMCs with peptide #10_short. To quantify B cells and monocytes, PBMCs were stained with antibodies specific for the markers CD3, CD14, CD11b, CD19 and HLA-DR and viability dye.

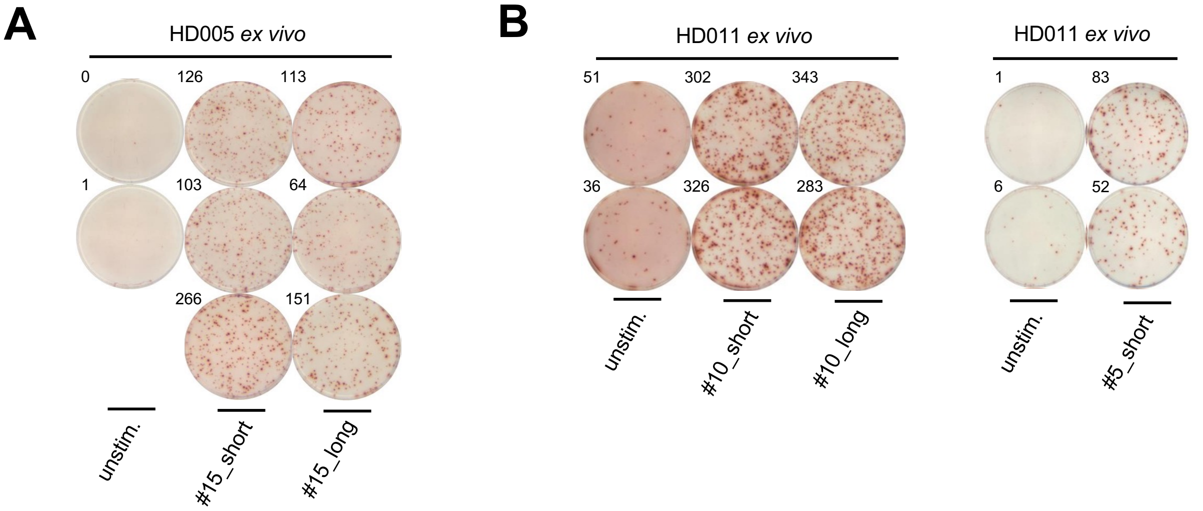

Supplementary Figure 3. *Ex vivo* IFNγ ELISpot assay responses in HD005 and HD011. **(A)** Well images from *ex vivo* stimulation of HD005 PBMCs with peptides #15_short and #15_long. Top-left numbers indicate the counted SFUs per 500.000 cells. **(B)** Well images from *ex vivo* IFNγ ELISpot assay after stimulation of HD011 PBMCs with peptides #10_short, #10_long and #5_short. Top-left numbers indicate the counted SFUs per 300.000 cells. Data come from two separate experiments.

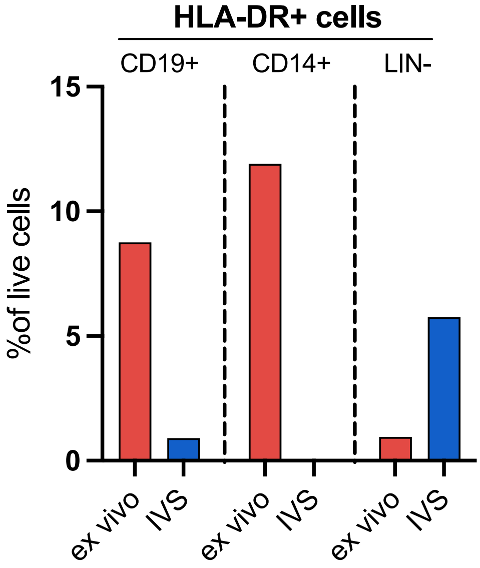

Supplementary Figure 4. Presence of APCs *ex vivo* or after IVS of HD011 PBMCs with peptide #10_short. LIN; linage

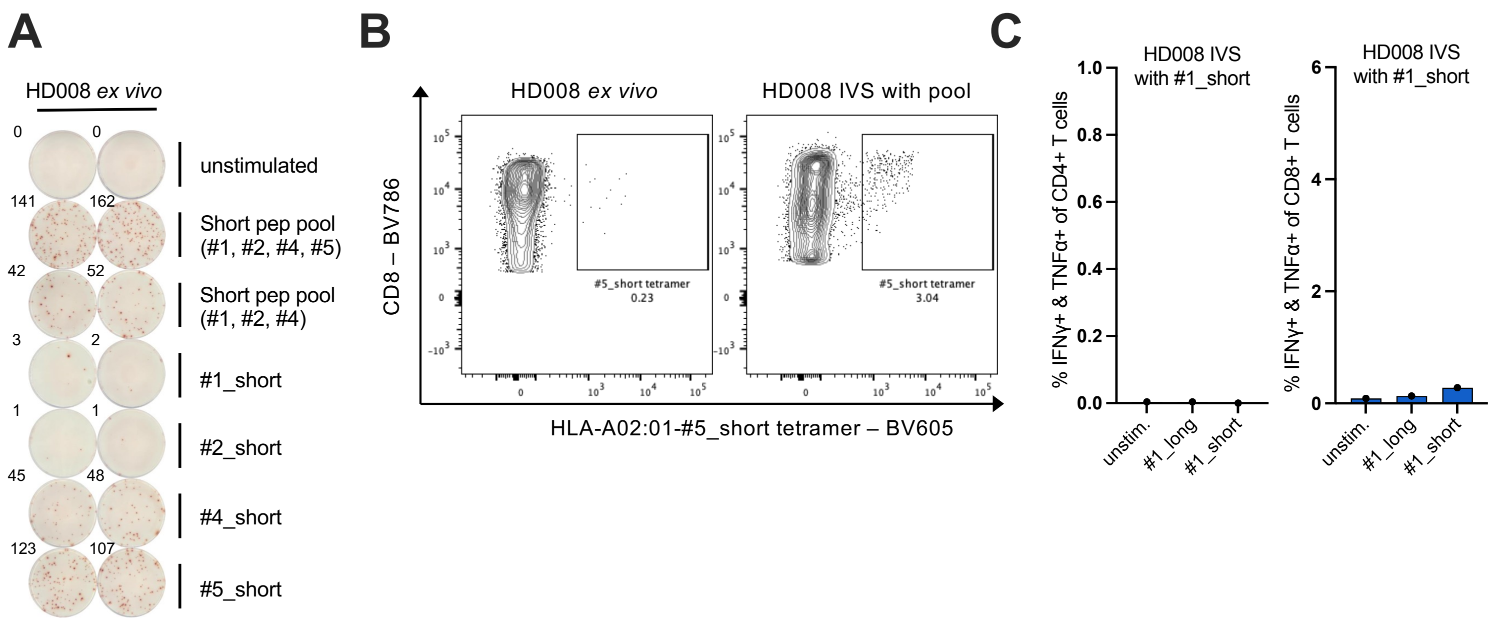

Supplementary Figure 5. Immune responses in HD008. **(A)** Well images from *ex vivo* IFNγ ELISpot assay after stimulation of HD008 PBMCs with a pool of the short peptides #1_short, #2_short, #4_short and #5_short, a pool comprising the three first peptides or with each of them individually. Top-left numbers indicate the counted SFUs per 400.000 cells. **(B)** BV605 fluorophore-labelled tetramers consisting of HLA-A02:01 alleles loaded with peptide #5_short were used to stain HD008 PBMCs either directly *ex vivo* or after IVS with the pool of the four viral peptides. **(C)** % IFNγ+ & TNFα+ within CD4+ and CD8+ T cells upon stimulation of PBMCs from HD008 with peptides #1_long and #1_short after IVS with peptide #1_short.

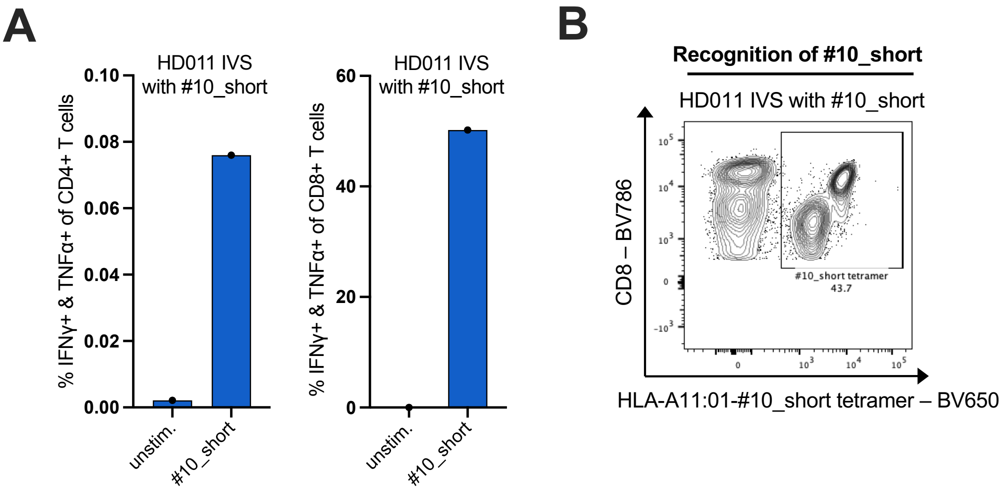

Supplementary Figure 6. Post-IVS immune responses in HD011. **(A)** % IFNγ+ & TNFα+ within CD4+ or CD8+ T cells upon re-stimulation of PBMCs from HD011 with #10_short peptide after IVS with the same peptide (n = 1 IVS culture). **(B)** BV650 fluorophore-labelled tetramers consisting of HLA-A11:01 alleles loaded with peptide #10_short were used to stain HD011 PBMCs after IVS with the same peptide.

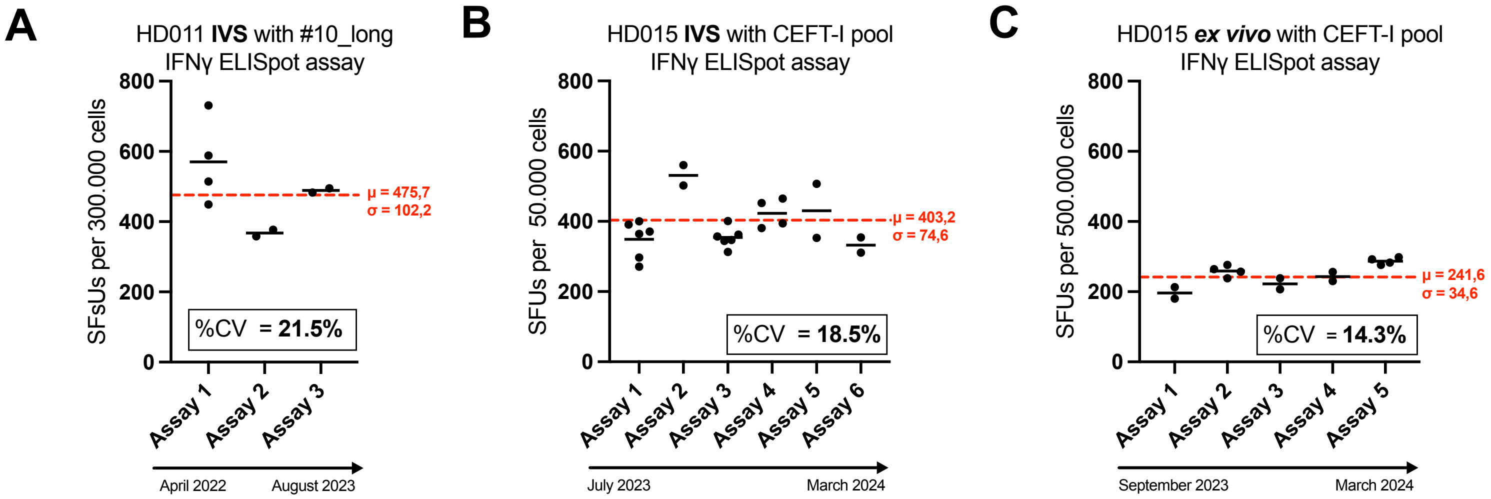

Supplementary Figure 7. Robustness of the alternative IVS assay. **(A)** HD011 PBMCs were expanded *in vitro* with peptide #10_long following the alternative IVS protocol and re-stimulated in an IFNγ ELISpot assay with the same peptide. Data represent raw SFUs per 300.000 cells from three independent IVS and ELISpot assays performed between April 2022 and August 2023. **(B)** HD015 PBMCs were expanded *in vitro* with the CEFT-I pool following the alternative IVS protocol and re-stimulated in an IFNγ ELISpot assay with the same pool. Data represents raw SFUs per 50.000 cells from six independent IVS and ELISpot assays performed between July 2023 and March 2024. **(C)** HD015 PBMCs were stimulated *ex vivo* with the CEFT-I pool in an IFNγ ELISpot assay. Data represents SFUs per 500.000 cells from five independent IVS and ELISpot assays performed between September 2023 and March 2024. Each dot represents data from a single well and the red dotted line represents the overall assay mean (μ). Inter-assay Coefficient of Variation (% CV) was calculated by dividing the standard deviation (σ) by the overall mean (μ) and multiplied by 100.
